# Impulsive action: emotional impulses and their control

**DOI:** 10.3389/fpsyg.2014.00518

**Published:** 2014-06-02

**Authors:** Nico H. Frijda, K. Richard Ridderinkhof, Erik Rietveld

**Affiliations:** ^1^Department of Psychology, University of AmsterdamNetherlands; ^2^Amsterdam Brain and Cognition, University of AmsterdamNetherlands; ^3^Department of Psychiatry, Academic Medical CenterAmsterdam, Netherlands

**Keywords:** action readiness, appraisal, control precedence, emotional impulses, impulse control, multiple emotions, impulsive action, motivation

## Abstract

This paper presents a novel theoretical view on impulsive action, integrating thus far separate perspectives on non-reflective action, motivation, emotion regulation, and impulse control. We frame impulsive action in terms of directedness of the individual organism toward, away, or against other givens – toward future states and away from one’s present state. First, appraisal of a perceived or thought-of event or object on occasion, rapidly and without premonition or conscious deliberation, triggers a motive to modify one’s *relation* to that event or object. Situational specifics of the event as perceived and appraised motivate and guide selection of readiness for a particular kind of purposive action. Second, perception of complex situations can give rise to multiple appraisals, multiple motives, and multiple simultaneous changes in action readiness. Multiple states of action readiness may interact in generating action, by reinforcing or attenuating each other, thereby yielding impulse control. We show how emotion control can itself result from a motive state or state of action readiness. Our view links impulsive action mechanistically to states of action readiness, which is the central feature of what distinguishes one kind of emotion from another. It thus provides a novel theoretical perspective to the somewhat fragmented literature on impulsive action.

## IMPULSIVE ACTIONS

The notion of *impulsive action* is often used but rarely specified. It refers to an action that is elicited by the appraisal of a perceived or thought-of object, event, or state of the world as pleasant or unpleasant, or as beneficial or harmful, and which action is apt to influence that object, event, or state of the world. The appraisal first gives rise to a state of readiness to establish, modify, sustain, or terminate the agent’s *relation* to the object, event, or state of the world concerned. That readiness then may induce an action that can implement the readiness’ aim. Actions are considered “impulsive” when and because they are not preceded by deliberation or the conscious representation of some action goal.

An impulsive action is thus defined as a non-deliberate action that serves the purpose of rendering one’s relation to the object, event, or state of the world more pleasant or less unpleasant. This definition answers the problem how an action can be purposive without being planned or premeditated. It is the appraisal that imparts the purpose by giving rise to a state of action readiness to change one’s relation to its object. It enhances interaction with what is appraised as beneficial or attracting. It decreases interaction with what is appraised as aversive or harmful, under the current situational conditions. *How* appraisal may impart purpose and elicit action readiness will be discussed in later sections on action readiness elicitation and on the neural bases of impulsive action.

Impulsive actions not only have a purpose or aim. They also have a certain strength or urgency. They generally possess some “impetus” – an inclination to *do* something. They owe that strength or urgency to being set for the forthcoming action, and its continuation until a particular end state has been reached, and resumption of actions in spite of interruptions and obstacles. “Impulsion” and “impetus” are terms dating from the middle ages and may seem old-fashioned, but we have a need for a concept that allows us to refer to the dynamic aspects of impulsive actions, and the energetics required to produce them. These various aspects have been caught under the designation of *control precedence* of ongoing impulsive actions and strivings (Frijda, 1986, 2007). Control precedence includes persistence of action over time until a particular end state has been reached, and resumption of actions in spite of interruptions and obstacles. For instance, one may continue making reproaches when one’s antagonist has already apologized for an offense. The number of knife stabs in violent marital fights is higher than in the average knife killing (Wolfgang, 1958). Impulsive actions entail selective attentional bias, overattending to what fits the impulsive action in course, and neglecting other information (e.g., Wiers et al., 2010). Impulsive actions also tend to be given precedence over other actions for which an elicitor happens to be present, and which the subject tends to disregard. These various dynamic features are intrinsic to the intent and impact of the impulsive action. The summed magnitude of the ratings of those dynamic properties over a past emotional incident correlates with ratings of how intense the emotion has been. That felt intensity can be considered as having been caused by the dynamic aspects of impulsive action (Sonnemans and Frijda, 1994).

As elaborated in earlier work, action readiness and changes in action readiness form the key to what we call “emotions,” since action readiness covers the variations in self-object relationships (Frijda, 1986, 2007). From this perspective, all impulsive actions qualify as emotional actions. However, not all emotional actions are impulsive. Many such actions and signs of a change in readiness for action aim at some premeditated goal and qualify as emotional on the one hand, but as deliberate rather than impulsive on the other. Moreover, many such deliberate emotional actions show restraint and control of actions that moderate the effects and magnitudes of impulsive actions.

## THE AIMS OF THE PRESENT PAPER

The present paper aims to convey the following views which, together, constitute a novel conjecture of impulsive action in relation to emotion regulation.

• First, impulsive actions are produced by motives to alter the current state of the world so as to approximate a more optimal state. These motive states themselves can be understood as *states of action readiness* (Frijda, 1986, 2007) that have a certain strength and urgency (i.e., impulsion).
• Second, these states of action readiness are instigated by how a perceived (or recalled or imagined) object or event is appraised by the individual: the degree to which it is pleasant or unpleasant, or expected to benefit or harm the agent. Given the elements of appraisal and action readiness, impulsive action is emotional action.
• Third, perceived, recalled, and imagined objects or events are often appraised in several different ways by the same individual, thereby eliciting multiple states of action readiness. These multiple states of action readiness may interact.
• Fourth: building on previous work (Mesquita and Frijda, 2011), such interaction may contribute to regulation of impulsive action, and hence to emotion regulation.

## STATES OF ACTION READINESS

Agents that are not acting still can be in a state of readiness for acting in a particular kind. They can be in “states of being set for presenting various patterns of behavior when the appropriate stimulus conditions in the external environment are available” (Morgan, 1943, p. 461; Gallistel, 1980, p. 321). The term *action readiness* was borrowed from Dewey (1895). Its analysis emphasizes that motive states are not representations of desirable states, but actual preparations for action, including the adoption of an attitude toward an object or event, from which an actual change of relationship with that object or event may emerge (Bull, 1951; Deonna and Teroni, 2012).

More precisely: states of action readiness presumably consist of subthreshold preparatory potentiations for establishing, enhancing, modifying, or maintaining some self-object relationship as well as potentiations of relevant coping skills. The self-object relationships that action readiness is concerned with manifest many different modes. Different modes of action readiness have different aims. One can be motivated to be and remain close to someone else; or to be hostile to someone; or to avoid some object or situation, or to relish in interacting with something delightful; or to entertain no relationship with any object whatever. Approaching and avoiding, attending, adopting a hostile stance, affiliation, dominance, submission, and effecting gratuitous interactions, as in joyful exuberance, are examples of such different motive states (Frijda, 2007). Certain modes of action readiness in fact correspond to certain common emotion names: hostility to anger, avoidance to fear, and submission to awe (Frijda and Parrott, 2011). “States of action readiness” include states of absence or disinclination for action. Along these lines we can say that a disinclination to act corresponds to apathy (Frijda and Parrott, 2011).

In any one given encounter with a particular object or situation in a particular mode of action readiness, a person can avail him- or herself of a number of different actions that have an equivalent effect (Frijda, 1986). When angry one can kick, shout insults, turn one’s back. These all are able to hurt or intimidate one’s opponent. When charmed by some person, one can kiss, offer flowers, or say endeared words: these all may result in drawing or keeping the charming person nearby. When running away in fear, one seeks to increase the distance to the danger source, but one may also hide behind a protective barrier, or throw an obstacle in front of the approaching threat. Selecting a particular action can only proceed when, consciously or unconsciously, a motive is established: the action should accomplish a particular modification in a particular self-object relationship, such as the riddance of an itch. Inversely, assuming an action to have a motive is an inference from observing a variety of equifunctional actions in a self-object interaction. A motive itself can be conceived of as being set to expect a particular input, along the lines proposed in predictive coding theory (Von Holst and Mittelstaedt, 1950; Friston, 2012; Clark, 2013).

Each state of action readiness can become manifest in various ways. They may show themselves in a variety of impulsive actions that share their aims. Action, and attention for the event and how it develops, tend to manifest features such as persistence and action power – features of control precedence. Actions implement relational motives that indeed modify the self-object relationship. But an aim may also appear in a mere fragment of action, a frown for instance, which can be understood as a fraction of an action of assembling effort; a slight backward tilt of the head betrays an attitude of keeping distance from the interaction partner. A state of action readiness may also be only felt by the agent, as a non-overt inclination, or experienced as a mental image of action (Ridderinkhof and Brass, submitted for publication). It finally may be evident only to the subject him- or herself in a covert cognitive action, such as thoughts of actions and of action-relevant objects. As such they may be felt and verbally identified. They may cause recurrence of the motives and urges in thought, as in thinking again and again of the person one fell in love with – even while tying one’s shoe-laces, or in noticing that one’s walks led by her or his house. They may lead to planning and execution of deliberate action at a later time, even when control precedence has decayed.

States of action readiness presumably consist of subthreshold potentiations of relevant coping skills, such as attentional orientations to target objects, setting of efference copies or of collateral discharges, perceptual expectancies, changes in motor cortex excitability (van Loon et al., 2010), and bodily preparations. The preparations may remain limited to mere potentiations of the neural network underlying the coping skills concerned, as yet without motor engagement. Action readiness may in such instances remain limited to readiness that is merely felt and not observable in overt behavior. Reports of feelings of different emotional urges (e.g., Davitz, 1964) indeed provide a major argument for the motive state or action readiness hypothesis.

## ACTION READINESS ELICITATION: EVENT APPRAISAL

What elicits action readiness? And what keeps it going? Emotions are aroused by events: events as directly perceived, or as described in a book or newspaper, or as observed on television or internet, or as related by a third person, or as recollected, or otherwise appearing in the mind, for instance in the form of thoughts. Motives and their states of action readiness (including autonomic responses) are elicited by events as these are appraised in a fraction of a second (Scherer, 2005; Frijda, 2007). *Appraisal* refers to the information that confers personal meaning to brute perceived events, which is drawn from environmental stimuli plus their temporal and spatial context, plus that drawn from actually interacting with the event, and that which is drawn from stored, associated information. The information can be of two kinds. It can pertain to what the event may do or offer to the individual (e.g., hurt him or her, carry satisfactions or signal likely coming satisfactions, deprive him or her from satisfactions). And it can pertain to “affordances” (Gibson, 1979): the action possibilities provided to us by the environment. This kind of information relates to event aspects that suggest possibilities for action that the event affords or does not allow.

Major appraisals result from interactions between event properties on the one hand and sensitivities of the individual on the other. They are often considered in terms of appraisal dimensions (Ellsworth and Scherer, 2003; Scherer, 2005). One such dimension of appraisal is the *valence* of an event: its intrinsic pleasantness or unpleasantness. Another is the individual’s appraisal of his or her ability to cope or deal with an event or its impacts, and the magnitude of the event’s impacts. Another again is the attribution of the event to some other person, to oneself, or to fate.

The most central appraisal is whether an event is appraised to promote or obstruct one or more of the person’s goals, needs, values, or more generally *concerns* (Ellsworth and Scherer, 2003; Scherer, 2005; Frijda, 2007). Concerns relate to what an individual cares about. Appraised concern pertinence is the condition for an event to operate as an emotional event. It provides the major basis for hedonic value: for pleasantness, unpleasantness, and desirability. In the absence of concern pertinence, there is no motive, no motive state, no change in action readiness; in short, no emotion. Concerns include everything that affects the presence, availability, or intactness of that which an individual cares about (cf. Frankfurt, 1988). They include conditions that are vital to survival, to attachments, but also values that the individual subscribes to, such as concerns for knowing the truth, for self-esteem, for being part of a social group, for well-being of others, for finding meaning in life, – concerns of which the motivating power may considerably vary from one culture to another (e.g., Schwartz, 1995).

An important kind of action readiness has as its aim to terminate or attenuate prepared or ongoing action. That kind of action readiness leads to actions that control or inhibit prepared or ongoing impulsive action. Control or inhibitory actions are triggered by the appraisal of expected as well as unexpected undesired consequences of one’s ongoing actions.

Appraisal processes largely proceed automatically and non-consciously (Lambie and Marcel, 2002). They can do so because received information, or information entering from memory can evoke a cloud of associations (Friston, 2012; Frijda, 2013). Yet, they are interspersed with conscious and deliberate efforts at exploring and articulating the informational content, whenever the automatic processes fail to give adequate guidance for further processing (Leventhal and Scherer, 1987). Not all available appraisal- and affordance-relevant information is processed. Scope and degree of attention are variable, and information pick-up is influenced by interests and perceptual habits. Control precedence of current action readiness, for instance, may appreciably restrict that pick-up (Easterbrook, 1959). In contrast, when affordances are relevant to the moment’s action orientation and concern engagement, they do influence the course of further processing (Rietveld, 2008, 2012).

Concern pertinence of an event, too, is largely appraised automatically. One often is not aware of why one loves, hates, likes, or dislikes (Nisbett and Wilson, 1977; Wilson, 2002). Concern pertinence of an event is mainly manifest after the facts of appraised event valence, emergence of action readiness, control precedence, and possibly subsequent impulsive action.

Appraisal – the entire pattern of values of the various appraisal aspects – is what generates the person’s state of action readiness: whether or not something has to be done with or about the self-object relationship, and if something has to be done, what has to be done, and with which urgency and control precedence.

But not only that. It also provides the motive for that state of readiness, and hence motivation for action. Appraisal identifies what relationship should be established or modified. One views an attractive person at the other side of the fence: the attractiveness primes aims for interaction and proximity, and the fence represents an obstacle that has to be dealt with, with a particular kind of action.

## THE AIMS OF IMPULSIVE ACTIONS

Impulsive action is situation- or event-elicited. That is: it is triggered by how the object or event is appraised. It is released by what the event or object suggests it may offer or may do to the agent, or may withhold from him or her. One is right in front of the relevant information for guiding one’s actions. Notice that no explicit goal representation is needed for such guidance. One faces an object or event that one is appraising. It is before one’s eyes or before one’s mind’s eye, and it remains there until one has finished acting. The action is guided by what Searle (1983) calls *intention in action*, and Dreyfus (2005) and Pacherie (2000, 2001) call *motor intention.* One can also just refer to it as an *intent*, evoked by what the object or event appears to promise or spell, in the way that relevant affordances do.

Crucially, adequacy and efficacy of impulsive actions scale with the availability of relevant skills or expertise. To the skilled badminton opponent, appraisal of even a well-concealed, crossed drop-shot may generate a relevant affordance for adequate non-reflective reaction which – without premeditation or further deliberation – renders a specific motive state, a readiness to act, an adequate action impulse. In contrast, a lack of relevant affordances may leave the novice stand “frozen” – that is, without an intent, without a concrete motive for action, without any action impulse.

Thus, an action is retrieved from one’s action repertoire that can implement the current motive; or one is constructed on the spot, primed by some affordance presented by the object or event. When hostile, seeing a gun lying around facilitates gun-using imagery (Berkowitz, 1974). When hostile and seeing a bicycle stand around, one may use that bicycle as a projectile and throw it at a police van during a street row. Retrieval from one’s action repertoire and *ad hoc* construction or improvised performance are motivated by current affect: by *directed discontent* (Rietveld, 2008). Adequate actions reduce directed discontent, by meeting the current motive of action readiness.

When encountering an event with emotional impact, plausible actions are retrieved from one’s action repertoire, with what the action should do – the motive – as the selection criterion. Every adult has a huge repertoire of relational action skills, both pragmatic and social one’s (Rietveld, 2012). Many of those skills have innate bases in body structure – clawing, biting, crawling, and grasping are prime examples in humans and other animals – that develop into true skills by pragmatic activities and play (e.g., Campos et al., 1996), that in turn form sources for perceiving affordances (Rietveld, 2012), and that can be adapted to other contexts than those in which they developed. One can bite to eat and one can bite to harm to implement a hostile action tendency.

The pragmatic and social actions alluded to are basic ones. They may require little information processing, and still often be effective. Approach, for instance, is an easy action that helps in implementing desire as well as intimacy. So-called emotional expressions are among those basic actions; they probably are prewired. Crying may well be viewed as an alarm call that often works in providing help upon an event that lead to the action readiness null-state of helplessness (Frijda and Parrott, 2011). Such basic expressions are informationally and computationally cheap, and thus afforded as non-reflective actions.

## ENGAGEMENT

Elicitation of action readiness requires a general condition in addition to appraisal: engagement. “S*trength of engagement* can contribute to experienced value through its contribution to the *experience of motivational force* – an experience of the intensity of the force of attraction to or repulsion from the value target” (Higgins, 2006, p. 439). It amounts to the capacity for interest, which enables information uptake and processing leading to appraisal taking place at all.

Specifying the nature of the condition of engagement depends on the theoretical interpretation of what that capacity of engagement or being interested consists of. It can be viewed as the capacity to energize motive states and subsequent action (Robbins and Everitt, 2007), and to invest perceived objects or events with *incentive value*, the power to want them (Berridge, 2007), or to learn that given stimuli predict that rewards or punishments will follow (Schultz, 2007).

All three – not very dissimilar – options are interpretations of the operation of neurotransmitters such as the midbrain dopamine system or, probably, of the dopamine/serotonin balance (Lewis, 2011). Drastic reduction of midbrain dopamine (in for instance Parkinson’s disease) renders the patient passive and unresponsive, and peaks in dopamine by contrast reflect enhanced motivation (e.g., Cools et al., 2010; Aarts et al., 2012) or craving (Lewis, 2011). Other neurotransmitters (serotonin, oxytocin, norepinephrine) appear to fulfill similar functions in regulating motivation in different motivational domains, such as those of withdrawal, contentment, affiliation, neural toning down, and curiosity (e.g., Tops et al., 2009; Cools et al., 2010; Jepma et al., 2010; Veening et al., 2010; Lewis, 2011; Murphy et al., 2011).

Near-total disappearance of action readiness can also occur because of more psychological conditions. We remarked above that there is no action readiness in the absence of concern pertinence. Concerns include everything that affects the presence, availability, or intactness of that which an individual cares about (cf. Frankfurt, 1988). On occasion, events may be appraised as unbearable, as urgently demanding change, but that no specific aim for producing change appears available. No meaningful action comes to mind, except “this has to end, in any possible way.” It would seem to be the background of urges toward suicide, or to sense of total incompetence or uncontrollability, which in our view is the background and structure of true anxiety (McReynolds, 1976). It occurs under torture and under threat of betraying comrades under torture (Lanzmann, 2009). Similar conditions would appear to be capable of inducing mental dissociation, or sense of estrangement and apathy (cf. Hilgard, 1977).

## PERCEIVED EVENTS MAY INDUCE MULTIPLE APPRAISALS

The preceding sections viewed appraisal as arousing action readiness, and action readiness as arousing some impulsive action, jointly with specific attributes of the event (as for instance its distance from the agent, the presence of shelter). However, emotional events are rarely simple. Encountered objects or events often can be appraised by a given individual in several ways at the same time. They thereby may generate several different states of action readiness, which may interfere or interact. The smell of roasted lamb chop may awaken desire, while simultaneously the thought of the poor killed lamb may elicit aversion. Appraisal of objects or events thus can give rise to multiple simultaneous changes of states of action readiness.

Multiplicity of emotion frequently occurs, since most situations present a multiplicity of affordances, and touch upon a multiplicity of concerns. Oatley and Duncan (1992), without extensive probing, found 31% of diary-reported emotions to be multiple emotion instances.

There is in fact nothing mysterious about several motive states being manifest simultaneously. They may show in the complexity of expressive behavior, as when a person looks at someone, but is not facing him or her in full; or as when meeting someone and mingling evidence of affection (a smile, say), invitation (opening the eyes more widely), and abandoning oneself, by adding a more erect stance. Such blends appear to closely mirror the momentary shades of readiness for further interaction. Similar sorts of blends appear when someone is tense, shows a defiant glance and clenches his or her fists, but also leans backwards in disengaging from direct personal interaction. Such motive state interpretations of expressive behavior can be experimentally validated. In several studies, subjects had to score expression pictures in terms of action tendencies, in which they obtained action readiness ratings that were about as consistent among raters as were their emotion label assignments (Tcherkassof et al., 2007).

In addition, emotion-inducing events frequently carry multiple meanings. They instigate several different appraisals. The death of a close loved one implies a loss but may also entail relief from care and worry. The two facts are appraised as sad and as not unpleasant, respectively. They may well incite different states of action readiness, such as respectively passivity and renewed openness for the world.

One and the same event thus may allow different appraisals to emerge almost simultaneously, even for the same individual. That constellation is common. Being treated unkindly by one’s spouse evokes one’s anger. But one may realize that one’s very emerging anger also may hurt one’s cherished spouse, with whom one is inclined to interact with consideration, if only for not spoiling one’s evening together. In fact, every novel element in perceiving an object or event is appraised. As long as one is attentive, one appraises what the event suggests it might offer, what it might require to cope with what the object might *do* to the agent, or to as yet obtain what it might refuse or yield. And all that appears to be within reach, since the agent is looking at the situation. And each newly perceived element may modify the current action readiness. When you see a child falling into the water, you appraise that event as urgently needing intervention, and you may impulsively jump after it, unless something new occurs that cuts one’s jumping short. Or the efforts spent in the impulsive action terminate the engagement, and action readiness gets into a lull.

## MULTIPLE EMOTIONS MAY INTERACT

Simultaneous motive states can be expected to interact. They in fact have to interact, since they have to share output channels: action provisions, attention resources, logistic support resources, and so forth. The interactions are required for coordinating the multiple emotions’ calls for action. Such coordinations lead to motive states, actions, and feelings that differ from those that would have become manifest when each emerged in isolation. Together, they result in mixed emotions or mixed feelings (Schimmack, 2001). For instance, affect is weakened or troubled when pleasure and pain occur simultaneously. Sense of ambivalence and vacillation appear, as extensively shown by Cacioppo et al. (2004), when unipolar rather than bipolar measures of affect were used (e.g., Wiers et al., 2005). True mixed feelings are observed in nostalgia, consisting of pain moderated by the happiness that was, together with pleasure moderated by the regret that it had gone (Bellelli and Saldarelli, 1990). Enhancement of pleasure and desire can be expected to occur when two sources of pleasure or desire happen to coincide: he or she is both bright and beautiful.

But what happens when multiple kinds of action readiness interaction depend upon their relative strengths. Net inclination and the power of action may become attenuated. It does, for instance, in approach/avoidance conflict such as examined by Miller (1959), where the animal stands still halfway. Or compromise or synthesizing action may be elicited when it is available in the individual’s repertoire, by having been acquired by socialization, with sharing as a solution of possession conflicts as an example (Campos et al., 2004; Mesquita and Albert, 2007). Or one mode of action readiness (e.g., withdrawal, by anticipation of shock) may override the other action readiness (e.g., approach to one’s infants; Richter, 1927), or sexual desire being overridden by approach of a powerful rival (De Waal, 1982), or by an overbearing or insensitive partner.

## MULTIPLE EMOTIONS CAN EXERT CONTROL

The interaction of multiple states of action readiness is illustrated in regulation of action, instigated by the concurrent appraisal of the expected consequences of one’s own impulsive actions (Gray and McNaughton, 2000). Anger is often held back by fear of retaliation or loss of affection. Fear of danger can be kept in check impulsively by shame or fear to appear sissy to others. Desire for drink or drugs may be tamed by the foresight of a hangover, of one’s upcoming loss of self-esteem, or one’s behavioral inefficiency.

The interaction of multiple modes of action readiness is one of the mechanisms of impulse regulation. One does not want to hurt one’s spouse, even if he or she just hurt you. Although very much liking to drink, one also does not want to become a useless or despised drunk. One wants to escape from the pains of abstention of smoking or drinking, – those stinging, breath-cutting urges that may feel like dying if not satisfied at once (Baker et al., 2004).

This implies that processes that regulate action are at least to some extent impulsive. These processes may not have regulation as their goal, but make way for concurrent or competing action readiness. One state of action readiness may happen to weaken or modify another one, such as action readiness for tender interaction on occasion happening to attenuate hostile approach, depending upon which of the two promises more benefit. We may suspect that in such constellations the emotion-regulating action readiness captures the action system non-reflectively and calls on action inhibition and override systems, relying on the same action control networks in the brain as engaged by other types of impulsive action (reviewed in the section on the neural bases of impulsive action).

Interaction between simultaneous motive states can also avail itself of a number of general purpose actions that presumably do not operate in goal-directed fashion, such as hesitation and postponing, preferring to fully or only partly engaging attention, being more or being less receptive for available information, such as responding to affordances.

There are many more automatic interaction processes: such as automatic inhibition as generated by anxiety elicited by unwanted impulses (Gray and McNaughton, 2000; Rothbart and Sheese, 2007) and by unwanted information, leading to shrinking back from exploration of threatening cognitive contents (Derakshan et al., 2007); and those that implement the range and degree of attention (Posner and Rothbart, 2007), from hypervigilance to deflecting attention, and the self-organization in the fusing of action tendencies, and in the feedback processes that contribute to action by ensuring coordination between activations (Lewis, 2005; Lewis and Todd, 2007). Thompson et al. (2008) detailed a number of such mechanisms, of different levels of complexity and informational involvement, down to vagal control (Porges, 2007).

## EMOTION REGULATION AND IMPULSE CONTROL

Action and impulse regulation are in fact largely driven by affectively steered states of action readiness. They to a large extent result from affective appraisals of action options, and the following of action-outcome preferences. Some impulses are best held in check until more appropriate times: when holding a cup of hot coffee, an itch may well trigger a scratching impulse, but one would do better to put the cup on a nearby table before giving in to the urge. In soccer, a jinx-movement by one’s direct opponent will manage to capture an impulsive reaction unless one can hold one’s horses and keep one’s eyes on the ball. The bases for such regulatory effects can be phrased more precisely. Emotions are moderated or controlled to the extent that one emotionally cares about the affective consequences of their being uncontrolled. *Caring* here means: grasped as contributing to concern satisfaction, or as avoiding to violate concerns. They are controlled, for instance, when one considers it worthwhile to behave in socially agreeable fashion, or when an addict considers it worthwhile to suffer the pains and efforts of abstention. One may consider it worth to suffer prolonged torture for not betraying a comrade, and even to commit suicide to prevent betraying. These are forms of regulation that Kuhl and Koole (2004) distinguish from simple self-control under the heading of *self-maintenance*. Satisfying the concerns involved in not betraying a comrade or in accepting to stop taking drugs or drinks evidently makes it worth to pay the costs: satisfying the concerns involved evidently outweighs the suffering of pains. The concerns satisfied by not betraying the comrade and by stopping drugs and drinks are concerns one accepts – that is, that one accepts to be concerned about.

That implies that such regulation also expands the range of acceptable action options, such as endeavoring to endure torture, or to accept the sufferings of abstention. It renders even seriously dealing with a serious temptation or addiction a matter of choice, as long as some energy for coping remains. Abandoning to temptations and abandoning self-maintenance are not always inevitable (Frijda, 2010; Lewis, 2011).

## NEURAL BASES OF IMPULSIVE ACTION

The outcome of appraisal directs one toward action that helps to optimize one’s relation to the world. But *how* does impulsive action become purposive? What are the neural mechanisms that support purposive impulsive action?

In order for anticipated action effects to be fine-tuned vis-à-vis what the action should accomplish, and hence allow for purposiveness of the action, a pragmatic anticipation of an action’s effect must entail the prediction of its sensory consequences (Ridderinkhof, in press). Such pragmatics consists of anticipations of exteroceptive and interoceptive sensation associated with the action (including its direction, extent, strength, and velocity, as well as the corresponding kinesthetic sensations; James, 1890). The pragmatic anticipation of some action effect can serve to retrieve, *deliberately or impulsively*, the particular action that gives rise to the particular sensory effects as associated with that action-effect (Herwig et al., 2007). The pragmatic anticipation of an action can be elicited through non-reflective priming by action affordances met and recognized along the way.

At the neural level of analysis pragmatic anticipations are neural activation patterns generated on-the-fly. Several brain circuits contribute to these patterns. The pragmatic anticipation of an action that serves to predict how the movement will unfold is formed in the posterior parietal cortex (PPC; Desmurget et al., 2009). Patients with apraxia resulting from damage to PPC have difficulty in generating pantomime performance of what one would stereotypically do with a hammer or comb (Clark et al., 1994), even though they can perfectly mimic the act when it is pantomimed by a model. The pragmatic anticipation of the action’s effects is mapped from PPC onto premotor and motor regions to activate the corresponding motor programs (Sirigu et al., 1996; Snyder et al., 2000). The neural activation patterns of pragmatic anticipation may in fact activate the descending motor pathways at subthreshold level (e.g., Jacobson, 1927; Tanaka et al., 2008). The pragmatic anticipation of an action’s effects thus awakens, at least incipiently, the actual movement which is its object, often without one’s awareness (Münsterberg, 1914 ). As we have proposed elsewhere (Ridderinkhof, in press), this change in action readiness (or incipient motor capture), incited by a pragmatic anticipation, provides a basis for forward models of anticipated sensory, kinematic, and muscular action effects, and may serve to prepare for effecting some action that reduces the discrepancy between our current state and the optimal state (Deonna and Teroni, 2012). The cerebellum receives direct feedback from several sensory modalities, allowing for the sensorimotor integration needed for on-the-fly adjustments as the action unfolds (Dietrich, 2008). The cerebellum has been proposed to be a crucial component in the state estimation process that combines information from motor efferent and sensory afferent signals to produce a representation of the current state of the motor system (Kawato, 1999; Miall et al., 2007). Thus, together, these circuitries may constitute an important circuit for anticipating the sensory consequences of the movement (Gerardin et al., 2000), and hence for rendering impulsive action purposive.

Frontostriatal circuits in the brain, capitalizing on the inferior frontal cortex, the pre-supplementary motor area (pre-SMA), and the basal ganglia, are engaged when impulsive actions are to be interrupted, aborted, suppressed, or overridden by more appropriate actions (Jahfari et al., 2011; Ridderinkhof et al., 2011), even when such action control is triggered unconsciously (van Gaal et al., 2008). When multiple pragmatic anticipations compete for bottom-up activation, the demands on action control are highest, and selecting the appropriate action engages stronger activation of the pre-SMA compared to when such bottom-up activation is absent. In these demanding situations of action conflict, the dorsolateral prefrontal cortex provides top-down guidance to action-selection areas (Hester et al., 2007), the pre-SMA engages response inhibition as an instrument of action selection (Swann et al., 2012), the inferior frontal cortex is recruited to aid in implementing response inhibition (Jahfari et al., 2011; Swann et al., 2012), and the basal ganglia keep all responses in check until further processing has converged on an action decision, and the final call is received from upstream: from the pre-SMA and other prefrontal regions (Jahfari et al., 2011). Indeed, impulse control deficiencies are observed in Parkinson’s disease (Wylie et al., 2012) and other clinical populations with problems in these frontostriatal circuits (Ridderinkhof et al., 2012; De Haan et al., 2013; Rietveld et al., 2013).

## CONCLUSION

Let us conclude by summarizing what can be drawn from this analysis.

First, impulsive actions are instigated by events that, by non-reflective processes of appraisal that have assessed the event’s relevance to the individual’s concerns, elicited some motive to establish or modify a self-object relationship. The ensuing state of action readiness drives and instigates impulsive action that might contribute to attaining the improved relationship.

Second, action selection when implementing the motive state in responding to events results neither from deliberate goal-directed action nor from overlearned habits, but from non-reflective action priming by affordances met and recognized along the way. Such priming elicits a pragmatic anticipation of the action’s effects, which entails the prediction of its exteroceptive and interoceptive sensory consequences. This allows for the evaluation and fine-tuning of anticipated action effects vis-à-vis what the action should accomplish, which renders the impulsive action purposive. Through efference copies or forward modeling, the pragmatic anticipation leads to a state of action readiness. As a result, impulsive action is by definition emotional.

Third, the perspective taken here denies the exclusivity of emotion regulation processes. There is no clear distinction between processes of emotion and processes of emotion regulation. In large measure, emotion regulation also proceeds impulsively: effortless, by the interaction of concurrent impulsive, emotional processes.

Fourth, viewing emotion regulation and impulse control at least in part as outcomes of the interaction of multiple states of action readiness is an alternative to viewing them as the outcomes of shifts of the operation of an “impulsive” processing system to a “reflective” processing system, in a dual processing fashion, as this was viewed by Strack and Deutsch (2004). They should not, in general, be seen as the outcomes of reason controlling emotion, or the rider controlling the horse. Quite on the contrary, our perspective accords with Hume (1739), in viewing reason as the slave of the passions. Reflective and deliberate processes seek to help solving impulsive dilemmas that impulsive processes alone are unable to solve.

Fifth, impulse control and emotion regulation can be expected to be served by enhancing relevant multiple emotions by focusing on non-obvious but relevant concerns, such as moral concerns and self-esteem.

## Conflict of Interest Statement

The authors declare that the research was conducted in the absence of any commercial or financial relationships that could be construed as a potential conflict of interest.
